# Monitoring of urinary iodine concentration in naval pilots: should iodine be supplemented or limited at coastal stations?

**DOI:** 10.1186/s40779-024-00511-0

**Published:** 2024-01-22

**Authors:** Jia Zeng, Qi Li, Xiang Lu, Dan-Dan Liu, Rong-Guan Jiao, Yan-Qing Jiang, Yan-Bing Liu, Wan-Qing Xu, Jun Ma, Guo-Li Gu

**Affiliations:** 1 Department of Aeronautical Diseases, Naval Medical Center, Naval Medical University of Chinese PLA, Shanghai, 200052 China; 2 No. 92853 Station Troops Hospital of Chinese PLA, Huludao, 125000 Liaoning China; 3 Department of Intensive Care Unit, Naval Medical Center, Naval Medical University of Chinese PLA, Shanghai, 200052 China; 4 Department III of Internal Medicine, Naval Medical Center, Naval Medical University of Chinese PLA, Shanghai, 200052 China; 5 Department of General Surgery, Air Force Medical Center, Air Force Medical University of Chinese PLA, Beijing, 100142 China

**Keywords:** Naval pilot, Thyroid, Urinary iodine

Dear Editor,

In recent years, there has been a significant upward trend in the detection rate of thyroid diseases among military flight personnel [1]. Thyroid diseases not only pose a serious threat to the health of flight personnel but also have negative impacts on the aviation safety and combat effectiveness of troops. It has been confirmed that the occurrence and progression of thyroid diseases are closely linked to dietary iodine intake. Studies have manifested that long-term excessive or insufficient iodine intake can lead to thyroid diseases [2, 3]. Currently, the relationship between the high incidence of thyroid diseases and iodine intake among military flight personnel remains unclear. In particular, the concentration of iodine in the diet and drinking water is usually higher in coastal areas than in inland areas [4], but whether such a high concentration affects the thyroid gland of naval flight crew at airport stations is yet to be determined. Therefore, it is of great significance to investigate the iodine nutritional status and spectrum of thyroid disease among naval pilots in coastal areas. In this context, this study was conducted to explore the correlation between iodine nutritional status and thyroid disease among military pilots based at a coastal flight station in China.

An investigation was first conducted on the dietary types and the use of iodized salt in the canteen of a coastal flight station. The results showed that the iodine concentration in the iodized salt provided at the canteen was 25 mg/kg (range: 18–33 mg/kg). The diet was reasonable, with no evidence of long-term excessive intake of seafood and other high-iodine foods. As only a small portion of the absorbed iodine accumulates in the thyroid tissue, approximately 90% is excreted through urine as urinary iodine. Consequently, monitoring urinary iodine concentration serves as a crucial indicator for assessing short-term iodine nutritional status and reflecting recent dietary iodine intake of flight personnel. As shown in Additional file 1: Table S1, this study enrolled a total of 183 naval pilots, including 131 fighter pilots with an average age of (31.0 ± 5.7) years and 52 helicopter pilots with an average age of (27.0 ± 5.7) years, along with 200 flying cadets without flight training who had an average age of (21.0 ± 1.0) years, and 889 ground crews with an average age of (25.0 ± 5.4) years. The iodine concentration in their morning urine was dynamically monitored, and it was found that the total urinary iodine concentrations of fighter pilots, helicopter pilots, flying cadets, and ground crews [(107.0 ± 39.8) μg/L, (113.5 ± 46.3) μg/L, (176.0 ± 66.5) μg/L, and (135.0 ± 57.0) μg/L, respectively] in the same coastal flight station were generally fall within the appropriate range (100–200 μg/L). However, there were statistically significant differences among the four groups (*P* < 0.001). Specifically, the urinary iodine concentration in fighter pilots and helicopter pilots were significantly lower compared with those of ground crews and flying cadets. Furthermore, there was a negative correlation between urinary iodine concentrations and age (*r* = −0.269, *P* < 0.001), as well as a positive correlation with flight time (*r* = 0.518,* P* < 0.001). Taken together, these data indicated an occupational association between urine iodine concentration and occupational positions, potentially attributed to the stress response in flight training.

Previous research has revealed that elevated levels of catecholamines and cortisol, induced by moderate to high stress, can lead to an increase in thyrotropin-releasing hormone and thyroid stimulating hormone, resulting in a temporary increase in thyroid hormone synthesis [5]. By examining the fastest heart rate, average heart rate, and stress index of pilots during flight training, it was observed that ship-based pilots exhibited significantly higher levels in terms of their fastest heart rate, average heart rate, and stress index compared with land-based pilots (Additional file 1: Table S2). Therefore, under equivalent iodine intake, the neuroendocrine system of pilots (particularly fighter pilots) will elicit psychological and physiological stress responses during air combat and aircraft carrier landing training in comparison with land-based takeoff and landing pilots. As depicted in Additional file 1: Fig. S1, the pilot experienced an overload axis in three dimensions to regulate flight attitude during takeoff and landing, resulting in a significant increase in heart rate and respiratory rate. The decrease in skin temperature is caused by sweating and evaporation under tension stress. This would result in an increased demand for iodine as their bodies would need to synthesize a greater number of thyroid hormones.

To explore the correlation between urinary iodine concentration and thyroid disease spectrum, thyroid function and morphology of naval pilots at coastal flight stations were examined. The results, as shown in Additional file 1: Table S1, indicated that 37.5% of flying cadets exhibited iodine excess. The median urinary iodine concentration of flying cadets with thyroid abnormalities was within the normal range (182.5 ± 63.2) μg/L, but significantly higher than that of flying cadets with normal thyroid [(161.3 ± 58.2) μg/L, *t* = 2.8, *P* = 0.005]. These findings suggest that excessive iodine intake may be associated with thyroid abnormalities in pilot cadets who did not undergo flight training in coastal areas characterized by high iodine concentration. This study also found that the relationship between urinary iodine concentration and thyroid function did not follow a simple linear regression model (*F* = 3.2, *P* = 0.075), which is consistent with a previous study [6]. Additionally, no significant correlation was observed between urinary iodine concentration and ultrasound results. However, in individuals with elevated levels of thyroid antibodies (anti-thyroid peroxidase antibody and anti-thyroglobulin antibody), ultrasound examination typically revealed heterogeneous changes, which are indicative of thyroiditis (Additional file 1: Table S3).

In conclusion, naval pilots residing in coastal areas with high iodine concentrations may continue to consume iodized salt as an annual iodine supplement. Particularly for navy pilots undergoing flight training, who may experience physical and psychological stress, it is advisable to appropriately increase iodine intake. Therefore, it is recommended that the monitoring of urinary iodine concentration be included in the short-term assessment of iodine nutritional status of naval pilots.

### Supplementary Information

**Additional file 1: Table S1** Urinary iodine concentration and thyroid disease spectrum in the four groups. **Table S2** Heart rate and stress status of ship-based and land-based pilots during flight training (mean ± SD). **Table S3** Urinary iodine concentration and distribution in subjects with thyroid dysfunction. **Fig. S1** Pilot’s physiological parameter during flight.

## Data Availability

All data supporting the results of this study are shown in this published article and supplementary documents. The datasets and materials used in this study are available from the corresponding author upon reasonable request.
