# Supplementary material for: Monitoring of urinary iodine concentration in naval pilots: should iodine be supplemented or limited at coastal stations?
Source: Mil Med Res. 2024 Jan 22;11:6. doi: 10.1186/s40779-024-00511-0 (PMC10802043; doi:10.1186/s40779-024-00511-0)
Supplement: Supplementary file 1 — Additional file 1: Table S1 Urinary iodine concentration and thyroid disease spectrum in the four groups. Table S2 Heart rate and stress status of ship-based and land-based pilots during flight training (mean ± SD). Table S3 Urinary iodine concentration and distribution in subjects with thyroid dysfunction. Fig. S1 Pilot’s physiological parameter during flight. [file 40779_2024_511_MOESM1_ESM.pdf]

Table S1 Urinary iodine concentration and thyroid disease spectrum in the four groups

Item	Pilots (n = 183)		Flying cadets (n = 200)	Ground crews (n = 889)	F/χ^2	P-value
	Fighter (n = 131)	Helicopter (n = 52)				
Age (year, mean ± SD)	31.0 ± 5.7	27.0 ± 5.7*	21.0 ± 1.0*#	25.0 ± 5.4*#&	107.3	0.000
Total urinary iodine concentration (μg/L, mean ± SD)	107.0 ± 39.8	113.5 ± 46.3	176.0 ± 66.5*#	135.0 ± 57.0*#&	47.0	0.000
Urine iodine concentration range [n (%)]						
0 – 99	55 (42.0)	20 (38.5)	22 (11.0)	197 (22.2)	50.1	0.000
100 – 200	72 (55.0)	30 (57.7)	103 (51.5)	570 (64.1)	13.5	0.004
201 – 299	3 (2.3)	1 (1.9)	69 (34.5)	106 (11.9)	93.8	0.000
≥ 300	1 (0.8)	1 (1.9)	6 (3.0)	16 (1.8)	2.3	0.518
Thyroid abnormalities [n (%)]	45 (34.4)	16 (30.8)	113 (56.5)	275 (30.9)	47.3	0.000
Urine iodine concentration in individuals with thyroid abnormalities (μg/L, mean ± SD)	121.3 ± 42.2	124.4 ± 52.3	182.5 ± 63.2*#	140.3 ± 64.1*&	34.8	0.000
Normal thyroid [n (%)]	86 (65.6)	36 (69.2)	87 (43.5)	614 (69.1)	47.3	0.000
Urine iodine concentration in individuals with thyroid normalities (μg/L, mean ± SD)	104.8 ± 45.5	108.8 ± 41.3	161.3 ± 58.2*#	132.4 ± 52.8*#&	35.6	0.000
Thyroid nodules [n (%)]	36 (27.5)	9 (17.3)	75 (37.5)	135 (15.2)	55. 9	0.000
Thyroid heterogeneous changes [n (%)]	3 (2.3)	1 (1.9)	6 (3.0)	25 (2.8)	0.3	0.961
Abnormal TPO-Ab and TG-Ab [n (%)]	8 (6.1)	2 (3.8)	24 (12.0)	100 (11.2)	6.1	0.105
Elevated TSH [n (%)]	1 (0.8)	1 (1.9)	2 (1.0)	8 (0.9)	0.6	0.896
Decreased TSH [n (%)]	1 (0.8)	1 (1.9)	1 (0.5)	10 (1.1)	1.1	0.768

*P < 0.01 vs. fighter pilots; #P < 0.01 vs. helicopter pilots; &P < 0.001 vs. flying cadets. *TPO-Ab* anti-thyroid peroxidase antibody, *TG-Ab* anti-thyroglobulin antibody, *TSH* thyroid stimulating hormone

Table S2 Heart rate and stress status of ship-based and land-based pilots during flight training (mean ± SD)

Group	Fastest heart rate (times/min)	Average heart rate (times/min)	Stress index during landing	Recovery ability index post-stress
Ship-based pilots (*n* = 52)	141.8 ± 13.0	115.3 ± 13.6	35.4 ± 13.4	5.2 ± 1.7
Land-based pilots (*n* = 113)	119.9 ± 14.9	101.9 ± 10.1	26.3 ± 10.6	3.0 ± 1.7
t-value	9.1	7.1	4.7	7.7
P-value	0.000	0.000	0.000	0.000

Table S3 Urinary iodine concentration and distribution in subjects with thyroid dysfunction

Item	Case (*n*)	Average age (year, mean ± SD)	Total urinary iodine concentration (μg/L, mean ± SD)	Urinary iodine concentration range [*n* (%)]			
				0 – 99	100 – 200	201 – 299	≥ 300
Thyroid nodules	255	29.0 ± 5.0	146.5 ± 64.6	21 (8.2)	164 (64.3)	53 (20.8)	17 (6.7)
Thyroid heterogeneous changes	35	26.0 ± 1.0	153. 2 ± 53.3	2 (5.7)	25 (71.4)	6 (17.1)	2 (5.7)
Abnormal TPO-Ab and TG-Ab	134	28.0 ± 3.0	171.5 ± 67.1	15 (11.2)	73 (54.5)	31 (23.1)	15 (11.2)
Elevated TSH	12	29.0 ± 2.0	154.5 ± 37.5	2 (16.7)	7 (58.3)	2 (16.7)	1 (8.3)
Decreased TSH	13	28.0 ± 1.0	102.4 ± 24.3	2 (15.4)	7 (53.8)	2 (15.4)	2 (15.4)
Normal thyroid	823	27.0 ± 5.0	132.7 ± 65.2	184 (22.4)	450 (54.7)	155 (18.8)	34 (4.1)
F/χ^2		8.3	10.3	61.0			

TPO-Ab anti-thyroid peroxidase antibody, *TG-Ab* anti-thyroglobulin antibody, *TSH* thyroid stimulating hormone

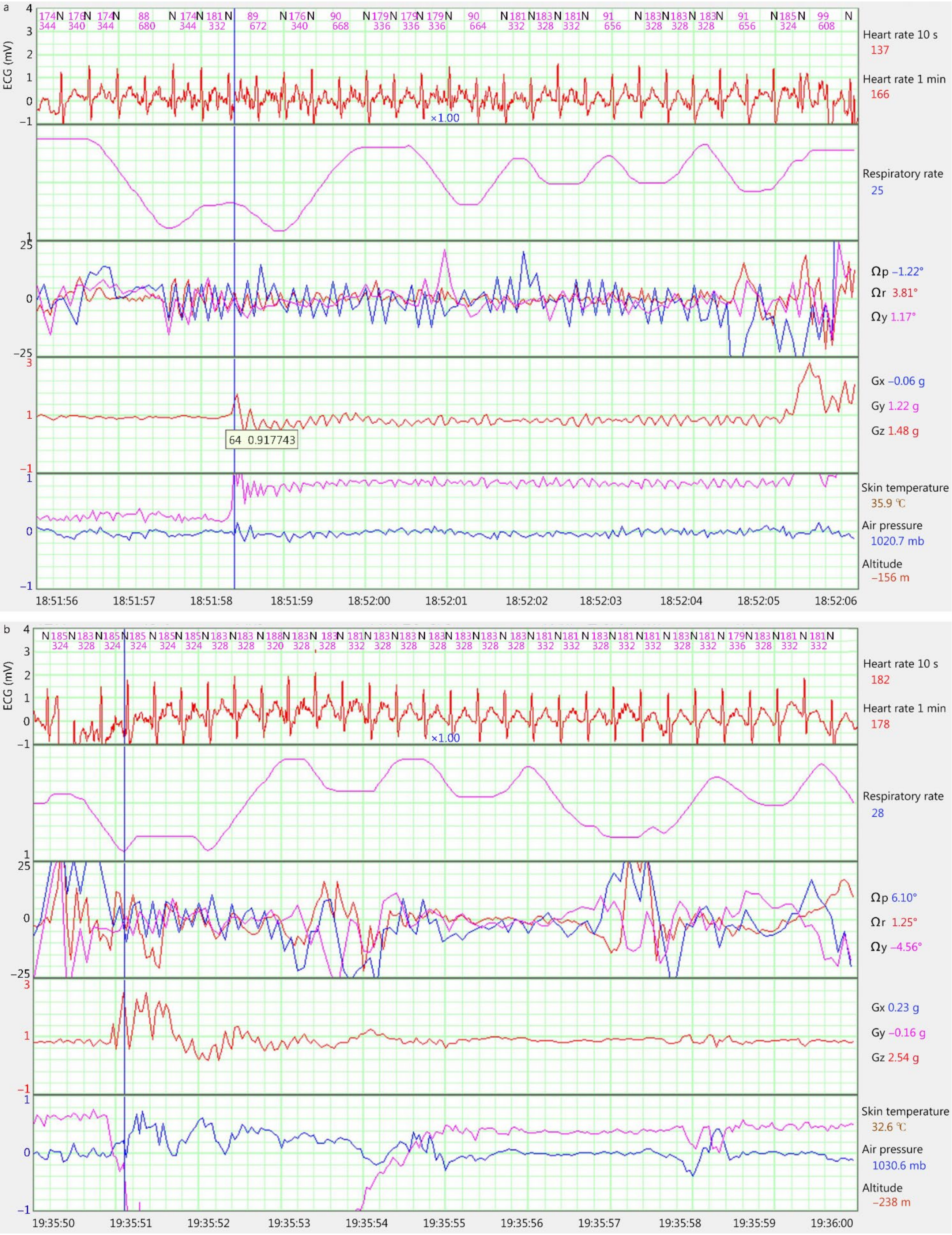

Fig. S1 Pilot's physiological parameter during flight. **a** Physiological parameter during takeoff. **b** Physiological parameters during landing. Heart rate 10 s represents the converted heart rate value every 10 s. Heart rate 1 min represents the converted heart rate value per minute. ECG electrocardiogram. The symbols Ωp, Ωr, and Ωr represent the flight attitude pitch, roll, and yaw, respectively. The symbols Gx, Gy, and Gz represent overloads in the x, y, and z-axis directions, respectively. Since altitude is converted from atmospheric pressure values, negative values appear
